# Assessing the Value of Further Investment in R&D Using Mixed Methods: A Case Study of Biosensor-Integrated Arteriovenous Grafts

**DOI:** 10.3390/jmahp13010001

**Published:** 2025-01-15

**Authors:** Samuel Owusu Achiaw, Neil Hawkins, Olivia Wu, John Mercer

**Affiliations:** 1 Health Economics and Health Technology Assessment (HEHTA), School of Health and Wellbeing, University of Glasgow, Glasgow G12 8TB, UK; neil.hawkins@glasgow.ac.uk (N.H.); olivia.wu@glasgow.ac.uk (O.W.); 2 BHF Cardiovascular Research Centre, University of Glasgow, Glasgow G12 8TA, UK; john.mercer@glasgow.ac.uk

**Keywords:** research and development, Health Technology Assessment (HTA), decision making, commercial viability, biosensing technology, renal dialysis, net present value

## Abstract

This study illustrates the utility of a mixed-methods approach in assessing the value of an example novel technology—biosensor-integrated self-reporting arteriovenous grafts (smart AVGs). Currently in preclinical development, the device will detect arteriovenous graft stenosis (surveillance-only use case) and treat stenosis (interventional use case). The approach to value assessment adopted in this study was multifaceted, with one stage informing the next and comprised a stakeholder engagement with clinical experts to explore the device’s clinical value, a cost–utility analysis (CUA) from a US Medicare perspective to estimate pricing headroom, and an investment model estimating risk-adjusted net present value analysis (rNPVs) to determine commercial viability. The stakeholder engagement suggested that it would currently be difficult to establish the current value of the surveillance-only use case due to the lack of well-established interventions for preclinical stenosis. Based on this, the CUA focused on the interventional use case and estimated economically justifiable prices at assumed effectiveness levels. Using these prices, rNPVs were estimated over a range of scenarios. This value assessment informs early decision-making on health technology R&D by identifying the conditions (including clinical study success, potential market size and penetration, market access strategies, and assumptions associated with CUA) under which investment may be considered attractive.

## 1. Introduction

Medical device development is a high-risk venture: it is capital-intensive, highly uncertain, may span many years, and, ultimately, technical success may not guarantee commercial success [1,2,3]. This compels developers, investors, and other stakeholders to make pivotal decisions in the initial phases of development based on how valuable the device is expected to be and if future returns will justify the required investment, given the associated risk. These decisions are particularly challenging in the initial phases of innovation due to the lack of clinical evidence [4]. Early Health Technology Assessment (HTA) or, as proposed by Bouttell et al. [5], “development-focused HTA (DF-HTA)” is increasingly being used to guide early decision-making in medical device development [6]. This paper illustrates the value of a mixed-methods approach incorporating both quantitative risk-adjusted net present value (rNPV) analysis and qualitative methods to explore clinical value alongside a cost–utility analysis (CUA).

This is illustrated using biosensor-integrated self-reporting arteriovenous grafts (smart AVGs) as an example technology. Currently, in preclinical development, the device is intended to use impedance spectroscopy to detect graft stenosis (surveillance-only use case) and electrically induce apoptosis, i.e., programmed cell death, to treat stenosis (interventional use case). The value assessment considers the two use cases and culminates in an estimation of the net present value of investment considering the full lifecycle from preclinical development to post-approval marketing.

An arteriovenous graft (AVG) is a synthetic conduit that is used to create a connection between a native artery and vein and is one of the three main types of hemodialysis vascular access [7,8,9]. Individuals with end-stage kidney or renal disease require life-sustaining renal replacement therapy (RRT), such as kidney transplantation, peritoneal dialysis, or hemodialysis [10]. Global estimates show that nearly 4 million people are on RRT, with hemodialysis being the most common modality, accounting for about 69% of RRT [11,12]. The hemodialysis procedure replaces the natural blood filtration function of the kidneys as a patient’s blood is passed through a dialysis machine, which removes waste and extra fluid [13]. The procedure requires vascular access (an AVG, an arteriovenous fistula or a central venous catheter) that allows an increased and continuous blood flow.

Current AVGs, such as expanded polytetrafluoroethylene (ePTFE) grafts, have high rates of stenosis and thrombosis, leading to low functionality, poor patient outcomes, and increased healthcare costs [9,10]. AVG stenosis is the narrowing of the lumen of the graft, often due to hyperplasia or increased proliferation of intimal cells of an adjacent blood vessel such as a vein [14]. This may result in the formation of a blood clot in the graft lumen, known as thrombosis, which leads to further occlusion of the graft lumen and consequently impeding the blood flow needed for hemodialysis [14]. A systematic review by Halbert et al. [15] found the mean primary patency rates of AVGs after one year to be 41%. Smart AVGs may help address the challenge of AVG stenosis and failure. Using electrical impedance spectroscopy, the device will be able to detect stenosis from vascular smooth muscle proliferation [16,17,18]. This will potentially enable continuous surveillance of graft patency, allowing for conventional interventions (such as angioplasty) to address stenosis before it becomes clinically apparent. This is referred to as the “surveillance-only use case”. In addition, the technology’s developers aim to incorporate into the device an electrically-inducing-apoptotic mechanism to re-establish patency when stenosis has developed [16,17,18]. This is referred to as the “interventional use case” (this comprises both surveillance and interventional functions with the smart AVG inducing programmed cell death, i.e., apoptosis, following the detection of stenosis by the surveillance capabilities). Both functions have been demonstrated in vitro [16,17,18]; however, the potential value of the device is yet to be established to inform developers and investors.

## 2. Methods

The approach to assessing the value of further investment in the development of the smart stent comprised:

1. A qualitative stakeholder engagement that explored the possible value of the two use cases of the device within current clinical pathways;
2. Based on the results of the stakeholder engagement, a cost–utility model was developed. The cost–utility analysis (CUA) estimated the incremental economic value and, hence, the potential price premium of the smart AVG compared to ePTFE grafts in the selected use case(s);
3. A risk-adjusted net present value (rNPV) analysis was conducted to identify the conditions under which investment in R&D may be regarded as commercially attractive based on the price premiums estimated from the cost–utility model and assumptions regarding market size, the likelihood of successful completion of each development stage, costs of capital, and development costs.

The approach presented in this study is multifaceted as it explores the potential value of a technology for both clinicians and patients through qualitative engagement and quantitative CUA, as well as the potential value for developers and investors through the rNPV analysis. Both the CUA and the rNPV model were constructed using Microsoft Excel for Microsoft 365 MSO (Version 2206 Build 16.0.15330.20260).

### 2.1. Qualitative Clinician Stakeholder Engagement

The stakeholder engagement comprised semi-structured interviews with four clinicians practicing in the US and UK (one interventional cardiologist, UK; one interventional cardiologist and peripheral endovascular specialist, US; one interventional nephrologist, US; and one consultant of vascular and transplant surgery, UK). Clinicians were purposefully approached based on recommendations by the technology developers and the clinicians’ experience in hemodialysis access and vascular surgery. Additionally, the US and UK are key target markets.

During interviews held over video conference (from March to July 2022), clinicians shared their opinions regarding whether and how the smart AV graft could offer clinical value. Following a description of the technology, discussions were structured around the following questions:

- Do you think the smart AVG in both surveillance and interventional use cases could provide significant clinical value for patients undergoing long-term hemodialysis?
- If so, how do you think the new technology might alter clinical pathways in the wake of vascular access complications?

### 2.2. Cost-Utility Analysis (CUA)

**Figure 1:**
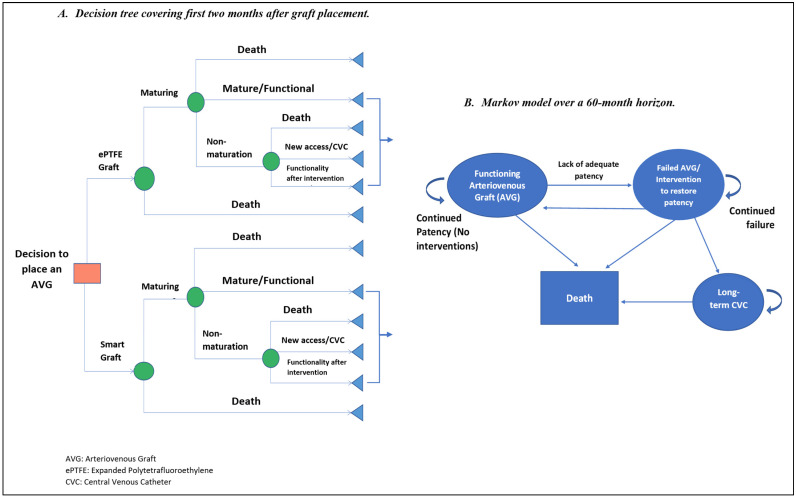
Decision Analytic Model.

Following the clinician engagement and a review of published relevant literature, a CUA was conducted. Given the results of the stakeholder engagement (see Section 3), the CUA and rNPV analysis were restricted to the interventional use case. The CUA estimated, given an assumed effectiveness level in graft failure reduction, the potential incremental net monetary benefit (INMB), i.e., the headroom price of the smart AVG over its comparator, ePTFE grafts. The INMB provides an estimate of the potential maximum price premium that the smart AVG might achieve over the ePTFE graft such that it could still be considered cost-effective at a given willingness-to-pay threshold. The CUA comprised a decision analytic model (Figure 1) with a decision tree modeling initial outcomes following graft placement and a Markov model of longer-term outcomes, including stenosis/failure (over a 5-year horizon).

The decision tree started with a decision node that considers the choice between the placement of an ePTFE graft and a smart AVG for incident hemodialysis patients. This was followed by three chance nodes for both arms of the tree. The first chance node was dichotomous, between maturing grafts and death. The next chance node follows the maturation stage and branches into non-maturation, mature/functional, and death. The final chance node is from the non-maturation branch (at which point there is an intervention or surgical revision due to non-maturation) and gives rise to the possibility of functionality (after intervention), new access (which is assumed in this study to be a central venous catheter (CVC)) and death. The outcomes of the initial graft placement were assumed to be the same for both the smart AVG and ePTFE grafts, as the smart AVG functionality was assumed not to affect the success of the initial implantation.

Patients in the functional graft state at the end of the decision tree entered the Markov model. The Markov model covered a 5-year (60-month) horizon with monthly cycles at the end of which patients may transition to a different health state. This time frame was deemed appropriate given the survival probability of hemodialysis patients (5-year survival probability estimated to be 43% of incident patients [19]). The model had four mutually exclusive health states: functioning graft state, failed graft state, CVC state (change in access after failure was assumed to be CVC), and death. Patients entering the functional graft state could transition to the failed state or death. Those in the failed graft state could transition back to functionality (following percutaneous or surgical interventions) or transition to the CVC state (if there was a failure to restore patency) or death. Within the CVC state, patients could only transition to the death state. Patients in the CVC state were assumed to be CVC-committed after repeated attempts to restore patency failed.

The decision tree and Markov model structures were taken from the study by Leermakers et al. [20]. A US Medicare/Medicaid perspective was chosen as the US is the current market focus of the developers and the technology currently has its patent filed in the US. An annual discount rate of 3% was applied to both future costs and outcomes according to the recommendations of the Institute for Clinical and Economic Review (ICER) [21]. A willingness-to-pay threshold of $100,000 per QALY was used in this study. ICER [21] recommends reporting health benefit price benchmarks using a range of $100,000 to $150,000 per QALY; taking a conservative stance, the lower end of this range was used. Parameters of the CUA, including the utilities, transition probabilities, and costs, were derived from the literature with details provided in Table 1 (further details are provided in the Appendix A). The decision tree and Markov model structures are illustrated in Figure 1.

### 2.3. Sensitivity Analysis of Graft Effectiveness

Given that the smart AV graft is currently in preclinical development, the effectiveness of the device in reducing stenosis is largely a matter of speculation. The model allows decisionmakers to estimate the potential price premium and expected discounted net present value based on their beliefs regarding likely smart AVG effectiveness.

The effectiveness of the smart AVG was hence expressed in terms of percentage reductions in the probability of transition from functioning graft to failure. The sensitivity analysis ranged from 0% to 80% effectiveness. The maximum effectiveness was assumed to be 80%, as the literature suggests that about 80% of graft failure results from stenosis and subsequent thrombosis [7,28].

### 2.4. Risk-Adjusted Net Present Value (rNPV) Analysis

The rNPV represents the expected net present value of the smart AVG in terms of discounted future net cash flows, taking into account the probability of success at the end of each stage of development and the eventual probability of the technology successfully getting to the market [29,30]. The rNPV model is linked to the CUA through the INMB, which is used as the unit cost for the smart AV graft. The main steps of the rNPV analysis are outlined below:

a. Defining the technology life cycle/rNPV time horizon

An appropriate time horizon should be selected to capture the relevant net future cash flows fully. For this study, a time horizon from concept development to 10 years post-market was used. A start year of 2023 was assumed for concept development, as this has already been underway. Development and approval were assumed to take 12 years [31]. A 10-year time horizon was assumed for sales based on remaining patent exclusivity and the likely development of competitor technologies. The total time horizon was, hence, 22 years (from 2023 to 2044), with 2023 serving as the present year for the estimated rNPV.

b. Identifying relevant (future) cash flows

Cash outflows comprised the costs of development and approval, the cost of goods sold (COGS), and selling, general and administrative expenses (SG&A). Development costs are outlined in Table 2. The COGS comprises all the costs directly incurred for the production of goods sold, including material and labor costs [32], and is assumed to be $100 per device. The SG&A was assumed to be 30% of sales revenue [33,34,35]. The interventional smart AVG was assumed to be a class III FDA device similar to currently used AVGs and based on classification descriptions by the FDA [36]. Accordingly, the average costs and timescale for the development and approval of class III devices were used [31]. This was estimated from a survey of over 200 medical technology companies [31]. Costs have been inflated to 2023 using steps outlined by ICER [21] (more details have been provided in the Appendix A). Probabilities of success by the stage of development were obtained from Sertkaya et al. [37].

Cash inflows comprised revenue from the sale of smart AVGs from 2035 to 2044. The unit price of the smart AVG was the INMB/headroom price obtained from the CUA. It should be noted that the INMB from the CUA is slightly understated as the technology cost of the ePTFE grafts was not included in the analysis. To rectify this, the COGS of the smart AVG was estimated as the additional manufacturing cost over the cost of the ePTFE graft. It was assumed that if a smart graft were to be launched, the cost of the ePTFE graft would reduce towards the average cost of production due to competition. The potential market size for AV grafts was estimated for each year from 2035 to 2044 based on OECD US population projections [38] and recent rates of AVG use in the US as reported by the US Renal Data System (USRDS) [19]. The mean incidence of hemodialysis cases per year from 2000–2019 was calculated to be 343.7 cases/million persons in the US population. From the USRDS reports, at the initiation of hemodialysis, most patients have a central venous catheter but transition to an AVG or AVF by 18 months. Recent reports show that after 18 months, about 16.9% (16.8% for those starting in 2017 and 17% for 2018) of patients initiating hemodialysis eventually end up with an AVG [19]. Using the average incidence of hemodialysis and US population projections by the OECD [38], hemodialysis cases for 2035–2045 were estimated. Of these estimates, 16.9% represented the AVG market size for each respective year. The market penetration was assumed to be 15% for the first year of sales and assumed to increase by 15 percentage points each subsequent year till 2039, after which the market penetration was assumed to stay at 75% till 2044. The total forecasted unit sales from 2035 to 2044 was estimated to be 130,368. The market size estimates and forecasted sales by year are shown in Table 3.

c. Identifying the “risk” in rNPV

The “risk” in the rNPV analysis refers to the risk of failure or, conversely, the probability of success of development. The probabilities of success estimated by Sertkaya et al. [37] were used in this rNPV analysis. Sertkaya et al. [37] estimated the probability of success from the nonclinical stages of development to the feasibility trial stage as approximately 46.9%. This probability was assumed to represent the probability from concept development to clinical safety trial, and the probabilities of each of the stages that this is comprised were assumed to be equal and derived accordingly. The probability of success for each of these three stages (concept development, clinical unit development, and IDE application) was hence estimated as 77.8%. The developmental stages and their respective probabilities of success are outlined in Table 2.

d. Selecting an rNPV discount rate

Expected future cash flows are discounted to account for the time value of money. The discount rate used reflects the opportunity cost of capital (OCOC). The opportunity cost of capital is the expected rate of return from the next best alternative investment of a similar risk level [37,39] and, hence, the minimum rate of return required for an investment to be seen as attractive. In practice, the discount rate may simply be the cost of capital, which is the rate of return needed to satisfy shareholders and debt holders [39]. The cost of capital may be expressed as a weighted average cost of capital (WACC), which is a weighted average of the cost of debt capital and the cost of equity capital [40]. However, as DiMasi et al. [41] and others [42] have pointed out, most financing in this sector is through equity, so the WACC is often dominated by the cost of equity capital. The cost of equity capital has been estimated to be between 9.2 and 11.4% for the medical device sector [43]. An OCOC/discount rate of 10.4%, an average by Sertkaya et al. [37] was used.

e. Calculating rNPV

The risk-adjusted net cash flow was calculated as the difference between the probability-weighted cash inflows and outflows. No cash inflows are expected until sales begin after premarket approval (PMA). The rNPV was computed using the following formula:

(1) rNPV=∑t=0nrNCt(1+disc)t

where rNC_t_ = risk-adjusted net cash flow for year t;

disc = discount rate;

*n* = end year.

This analysis only used present costs and future costs with 2023 as year 0. Past costs may be incorporated in the calculation, with *t* assuming a negative figure, which effectively increases rather than discounts as the denominator ((1 *+ disc*)*^t^*) takes on a value of less than 1. Past costs will also have to be inflated to the present year before being included in the analysis. An rNPV greater than zero implies that the expected return on investment is greater than the (opportunity) cost of capital over the duration of the project and, hence, potentially profitable/commercially viable.

f. Sensitivity Analysis (rNPV)

A two-way sensitivity analysis was conducted using an Excel data table. rNPVs were estimated for varying levels of smart AVG effectiveness and cumulative probabilities of success. For the two-way analysis, it was necessary to define the probability of success of each of the stages of development as a simplified function of the cumulative probability. It should be noted that different individual probabilities of success can accumulate to the same cumulative probability. Given that the development costs are weighted by the probability of success for the respective stage, it was necessary to ensure that the weighting for the stage costs retained their proportionality even when the cumulative probability is altered. As almost all stages of development had comparable probabilities, i.e., ranging between 75% to 80%, they were assumed to be equal. The probability of success for the clinical safety study used in the base–case rNPV model was, however, 48%. To obtain a similar probability as the other stages, this stage was split into three, each with a probability of success of 78.30%. With this, there were eight stages of development, from concept development to premarket (PMA) approval, compared to six in the base–case model. Following this, the cumulative probability could be defined as a function of the individual probabilities with the equation:

(2) *X* = *y*^8^

where

X = the cumulative probability;

y = the individual probabilities of each stage of development.

Using this equation, the individual probability of success was 78.03% which cumulates to 13.75%. Table 4 shows the changes made to the probabilities of success for the sensitivity analysis.

## 3. Results

### 3.1. Findings from Qualitative Research

From the qualitative engagement, the relevant use cases for the smart AVG were identified. Clinicians expressed views that the smart AV graft, as a whole, had the potential to improve clinical outcomes. The views, however, became divergent when the surveillance and interventional use cases were considered separately. While the interventional use case would be expected to provide value by reducing interventions and improving long-term graft patency, the value of the surveillance-only use case was unclear.

“…if the device can prevent these interventions, that would be impactful”—(Clinician No. 3).

Firstly, as expressed by the clinicians, the value of AVG surveillance is debatable, with surveillance remaining the judgment of clinicians rather than on established guidelines. In the US, for instance, it was mentioned that some centers may monitor patients with well-functioning grafts with physical exams and conduct further investigations, such as Doppler USG, where necessary. Patients with regular graft access dysfunction may, at their clinician’s discretion, have more frequent Doppler USG surveillance. This practice seems parallel to the 2019 Clinical Practice Guidelines by the Kidney Disease Outcomes Quality Initiative (KDOQI) [7]. These guidelines [7] recommend regular monitoring (by physical examination) but fail to recommend routine surveillance (through imaging for stenosis, measurement of access blood flow, or pressure monitoring) due to the low quality of supporting evidence. Importantly, the current guidelines [7] do not recommend pre-emptive interventions such as angioplasty for stenosis without associated clinical indicators (preclinical stenosis), as this has been shown to make no significant improvement in graft longevity. This is reiterated by the UK Kidney Association in their guidelines [44]. From the interviews, most clinicians mentioned that surveillance is unlikely to lead to interventions if there are no clinical signs present. It was further explained that given that some level of stenosis may be observed in normally functioning grafts (in some cases, up to about 50% or more of luminal narrowing), treatment may not be indicated in the absence of clinical signs.

It was, however, highlighted in the interviews that the evidence upon which the various guidelines have been based had several limitations.

“The RCTs on surveillance are all critically flawed and of little benefit”—(Clinician No.4).

Some of these limitations have been discussed by Kingsmore et al. [45], who highlighted the limitations in the study designs, including issues around study populations. The authors also described that up to 90% of significant stenosis before the development of thrombosis do not have reliable warning signs that may be picked up during monitoring, emphasizing the possible value surveillance could offer [45]. The authors [45] mention that stent grafts and drug-eluting balloons may be useful as pre-emptive interventions following surveillance with possible cost reduction and increased graft longevity. In such instances, surveillance could offer considerable value. However, according to current guidelines [7,44], evidence to support such pre-emptive interventions following surveillance remains inadequate. Additionally, DePietro et al. [46] have emphasized the need to appropriately time interventions for graft stenosis as angioplasty (the primary treatment) has been hypothesized to lead to barotrauma and vascular injury, instigating further neointimal hyperplasia, restenosis, and consequently, a vicious cycle of restenosis and recurrent interventions.

So, within the current clinical context, due to the lack of clearly defined interventions for preclinical stenosis, active surveillance is unlikely to significantly alter current practice and outcomes, and hence, the value of the surveillance-only use case remains uncertain. Following this, the CUA and rNPV analysis focused on the interventional use case for the smart AVG.

### 3.2. CUA Results

The CUA showed that graft failure after an initial successful implant accounted for a discounted loss of 0.43 life years and 0.33 QALYs and a cost of $17,207 over the modeled time horizon. The CUA was conducted at varying rates of effectiveness of the smart AV graft. At the lowest level of effectiveness tested (5%), an INMB of $1600 was obtained. At the peak of effectiveness (80%), the INMB was $31,841. The results of the CUA are shown in Table 5. Model validation is described in the Appendix A.

### 3.3. rNPV Analysis

The total discounted expected cost for development and approval processes was $33,282,266. An effectiveness level of 42% and a unit price of $14,903 was necessary to obtain a positive rNPV for a cost-effective smart AVG. Table A1 in Appendix A shows the results of the rNPV analysis for an assumed level of smart AVG effectiveness. Table 6 illustrates a two-way sensitivity analysis as the cumulative probability of success and the level of smart AVG effectiveness are varied.

## 4. Discussion

This study illustrates how a mixed-methods approach interlinked with a risk-adjusted NPV analysis may be used to explore the potential clinical and economic value together with the commercial viability of a novel technology. This approach draws on the strengths of various methods to estimate value in a multifaceted manner and is particularly suited to technologies at technology readiness levels (TRL) 2–6 [47]; in this example, the novel technology was in preclinical development (TRL 4).

By beginning the assessment with a qualitative engagement with relevant stakeholders/potential users of the technology, the use cases of the technology likely to be more valuable were identified, and subsequent analyses were tailored accordingly. Engaging diverse stakeholders is valuable as it allows for nuance and the consideration of important information that may not be readily available in published literature or obtained from clinical trials [48,49]. Clinician engagement has been mentioned by Smith et al. [50] to be of particular significance in medical device assessment, given the nature of medical device innovation. The authors note that medical device innovation has historically been technology-driven rather than by clinical need. Accordingly, the focal point has often been developers trying to find a way to translate technologies developed in other sectors, such as electronics or physics, into problem-solving medical devices [50,51]. That is to say, looking for the right locks (clinical problems) that may be opened by these new technological keys [50,51,52]. Also, given the lack of well-defined guidelines on pre-emptive interventions for stenosis, engagement with clinicians with different but relevant expertise was crucial in establishing current management practices and allowing for practical comparators in the assessment of the device. While developers of the smart AV graft are applying the technology in other disease areas, such as coronary artery disease [16,17], clinicians also mentioned that the surveillance function of technology could be beneficial in the management of conditions such as carotid artery stenosis where pre-emptive interventions may significantly improve outcomes.

The next step of the assessment was the CUA, from which threshold premiums for the interventional smart AVG could be estimated based on assumed levels of effectiveness. Described as a “headroom” analysis, this method of pricing is value-based, relying on a buyer’s willingness to pay for additional health value created, and has been used in other DF-HTA studies [2,6,53,54]. In the assessment of new technologies, the estimates of achievable price may be derived from cost-effectiveness modeling, by “payer” interviews, or by reference to comparable technologies. Cost-effectiveness modeling (CEM) may be most relevant to jurisdictions where it is an integral component of the reimbursement process (e.g., Australia, Canada, UK, Taiwan). However, CEM may still have predictive value in other jurisdictions. This study takes a US perspective where CEM, although generally not an integral component of most reimbursement processes, cost-effectiveness analyses, such as those conducted by ICER, may influence reimbursement processes [55]. According to ICER, more than 75% of private insurers and Pharmacy Benefit Managers reference their analyses [55]. It should be noted that the relevance of acceptable prices estimated through CEM is not binary. Even in jurisdictions where CEM is an integral component of the reimbursement process, other factors may be relevant, and CEM estimates may be contestable. Hutchings [56] highlights some of these factors, including the price of the comparator product, disease rarity, severity, and unmet need, as well as patent and marketing exclusivity. The effect of these factors may be complex or circuitous. Drummond et al. [57], for example, highlight the effect of comparator prices in the reappraisal of drug-eluting stents by the National Institute for Health and Care Excellence (NICE). Following the release of drug-eluting stents, their comparator, bare metal stents, fell in price [57]. Subsequent evaluations found drug-eluting stents to not be cost-effective as the incremental costs had increased even though incremental effectiveness remained the same [57]. Even so, CEM may provide a useful early indication of acceptable prices at a relatively low cost compared to “payer” interviews.

The final step in the assessment was the rNPV analysis using the estimated headroom prices from the CUA. For manufacturers and investors, the success of a medical device hinges on its commercial viability, i.e., the device’s ability to be competitive and profitable in the market [2,3]. Estimates of expected net returns in the early stages of development are beneficial as they guide decisions on further development, investment, market access, and strategy [1,2,3]. The rNPV analysis, while similar to other net cash flow approaches such as the NPV, incorporates the estimated probability of developmental success resulting in a more detailed and nuanced valuation. Although rNPV analysis, unlike real options analysis, may not be able to account for future uncertainty, volatility, and conditionalities, it is less challenging to implement and, as used in this study, aims to identify conditions under which technology may be commercially viable rather than provide definitive estimates of value. The rNPV model also illustrates the high-risk nature of medical device development. It is notable that although profit margins may be high if product development is successful, in the rNPV estimation, future revenues are heavily discounted both due to the high probability of failure and due to time discounting of future revenues (estimated as 82% in the current model). This is somewhat offset by the time discounting of development costs (estimated as 32% in the current model), but these costs are not as heavily discounted as revenues as they are more certain to be incurred and occur earlier. It is perhaps not surprising that many technologies in early development face the “valley of death”, a shortage of capital to fund the transition between preclinical and early clinical development [58].

### Limitations

This study had several limitations that are inherent to DF-HTA studies. An important limitation in this regard is the lack of evidence. The lack of evidence and resulting uncertainty, while common in most HTA studies, is more so in DF-HTA as the technologies being assessed are in development and may have no clinical trial data or evidence [5]. Bouttell et al. [1,5] have suggested bridging this gap through, among others, expert stakeholder engagement and the use of evidence from comparator technologies, as has been performed in this study. However, these methods may be less reliable than evidence collected from well-conducted clinical trials. Another limitation is associated with the clinician stakeholder engagement, which, while useful, involved only four clinical experts from two countries (the US and the UK) who were selected based on the recommendation of the technology developers. Consequently, conclusions drawn from the engagement may not be generalizable to other clinical settings. A further limitation was that the CUA assumed a simplified clinical pathway along with a deterministic decision analytic model, and as such, findings may not be applicable to more complex clinical pathways. Nevertheless, given that this study assesses the value of the technology in its very early stages with limited resources, these approaches were deemed acceptable, with the expectation of more generalizable and more complex analysis as more data and evidence on the new technology becomes available. A final limitation is the use of a single cost of capital in the rNPV calculations. Given the potential to crystallize value at interim stages of development by selling or licensing an asset and the considerable variation in capital requirements at different stages, it might be more appropriate to employ varying costs of capital (by stage of development) to reflect the variation in risk over time.

## 5. Conclusions

This multifaceted approach expansively assesses the value of new technologies by identifying the combination of conditions (such as clinical study success, appropriate discount rates, potential market size and product penetration, and assumptions associated with CEM and reimbursement) under which investment in R&D may be regarded as commercially attractive. For the example technology, the smart AVG, this assessment suggests that the “interventional use case” of the device may be clinically valuable as a hemodialysis access option, but this would depend on several unknown factors for which assumptions have presently been made. As medical devices, much less like drugs, undergo innovation throughout product development, trials, and even post-marketing [51,57], this study’s approach should be regarded as a dynamic and iterative one, allowing for the incorporation of more accurate evidence as a device progresses through its innovation.

## Figures and Tables

**Table 1:** Parameters for the CUA.

Parameter	Description	Value	Data Source (s)	Comments
Costs (2023$)	AVG (ePTFE placement)	$4641.19	Al-Balas et al. [22]	Reimbursement prices. Includes professional and facility fees.
	Intervention/Revision after non-maturation of graft	$4593.26		
	CVC placement	$896.97		
	Monthly cost for failed AVG state (monthly cost of interventions for failed AVG)	$647.62	Al-Balas et al. [22]	Source provides the median annual vascular access-related cost for AVG for 2015. Inflation applied, and yearly cost is converted to monthly cost.
	Monthly cost for CVC state	$2730.18	Al-Balas et al. [22]	Median annual vascular access-related cost (Percutaneous and Surgical Procedures, and Hospitalisation for Bacteremia, 2015).
Utilities	Functioning AVG (both ePTFE and Smart Graft)	0.6839	Wyld et al. [23], Scheetz et al. [24] Locham et al. [25]	
	Failed AVG (both ePTFE and Smart Graft)	0.5939	Brothers et al. [26], Scheetz et al. [24], Locham et al. [25]	-
	CVC	0.6526	Xue et al. [27], Brother et al. [26], Scheetz et al. [24]	
Transition Probabilities	Death after procedure	0.022	Leermakers et al. [20]	For the decision tree, probabilities, costs, and outcomes were assumed to be the same for both ePTFE and Smart AV graft arm.
	Death during maturation	0.054		
	Mature/functional (after maturation)	0.772		
	Death after non-maturation	0.011		
	New Access/CVC after non-maturation	0.065		
	Functionality after intervention (post-non-maturation)	0.076		
	Functioning graft state to continued function *	0.932 (for ePTFE grafts)	Leermakers et al. [20]	* For the Markov model, besides the transition from “function to failure” and “continued function”, all other transitions and corresponding probabilities and costs were assumed to be the same for ePTFE and Smart AV graft.
	Function to failure *	0.057 (for ePTFE grafts)		
	Function to death	0.011		
	Failed graft state to continued failure	0.280		
	Failure to functioning	0.533		
	Failure to CVC	0.120		
	Failure to death	0.067		
	CVC State to Continued CVC	0.974	Xue et al. [27]	
	CVC to death	0.026		

**Table 2:** Costs and probabilities of success of development.

Stage of Development or Approval	Cost	Probability of Success (by Stage)	Cumulative Probability	Duration (in Years)	Elapsed Time at the Beginning of the Stage
Concept Development	$12,277,791	77.8%	77.8%	3	0
Clinical Unit Development	$11,050,012	77.8%	60.5%	2	3
Investigational Device Exemption (IDE) Application	$13,505,570	77.8%	47.0%	1	5
Clinical Safety Study	$11,050,012	48.0%	22.6%	1	6
Pivotal Clinical Trial	$50,338,943	75.7%	17.1%	3	7
Premarket (PMA) Approval Process	$17,188,907	80.5%	13.7%	2	10

**Table 3:** Market size estimates by year.

Year	Market Size Estimate	Estimated Market Penetration	Forecasted Number of Sales
2035	21,195	15%	3179
2036	21,300	30%	6390
2037	21,403	45%	9631
2038	21,504	60%	12,902
2039	21,602	75%	16,201
2040	21,698	75%	16,274
2041	21,793	75%	16,344
2042	21,885	75%	16,414
2043	21,976	75%	16,482
2044	22,066	75%	16,550
Total			130,368

**Table 4:** Probabilities of success in base–case and two-way sensitivity analysis.

Base–Case Analysis			Two-Way Sensitivity Analysis		
Stage of Development or Approval	Probability of Stage Success	Cumulative Probability of Success (at End of the Stage)	Stage of Development or Approval	Probability of Stage Success	Cumulative Probability of Success
Concept Development	77.75%	77.75%	Concept Development	78.03%	78.03%
Clinical Unit Development	77.75%	60.45%	Clinical Unit Development	78.03%	60.89%
IDE Application	77.75%	47.00%	IDE Application	78.03%	47.52%
Clinical Safety Study	48.00%	22.56%	Clinical Safety Study 1	78.03%	37.08%
Pivotal Clinical Trial	75.70%	17.08%	Clinical Safety Study 2	78.03%	28.93%
PMA Approval Process	80.50%	13.75%	Clinical Safety Study 3	78.03%	22.58%
			Pivotal Clinical Trial	78.03%	17.62%
			PMA Approval Process	78.03%	13.75%

**Table 5:** Results of CUA at various levels of effectiveness.

Effectiveness Level of Smart AV Graft	Monthly Probability of Transition from Functional Smart Graft to Failure	Incremental Cost ($)	Incremental QALYs	INMB/Maximum Price Premium ($)
0%	0.0570	$0.00	0.0000	$0.00
5%	0.0542	−$537.40	0.0106	$1599.57
10%	0.0513	−$1090.64	0.0215	$3242.81
15%	0.0485	−$1660.34	0.0327	$4931.29
20%	0.0456	−$2247.10	0.0442	$6666.62
25%	0.0428	−$2851.59	0.0560	$8450.50
30%	0.0399	−$3474.48	0.0681	$10,284.70
35%	0.0371	−$4116.47	0.0805	$12,171.05
40%	0.0342	−$4778.31	0.0933	$14,111.47
45%	0.0314	−$5460.78	0.1065	$16,107.97
50%	0.0285	−$6164.68	0.1200	$18,162.64
55%	0.0257	−$6890.87	0.1339	$20,277.66
60%	0.0228	−$7640.22	0.1482	$22,455.31
65%	0.0200	−$8413.67	0.1628	$24,697.96
70%	0.0171	−$9212.18	0.1780	$27,008.12
75%	0.0143	−$10,036.78	0.1935	$29,388.38
80%	0.0114	−$10,888.51	0.2095	$31,841.45

**Table 6:** A two-way sensitivity table showing rNPV as smart AVG effectiveness and cumulative probability of developmental success are varied.

	Level of Smart AVG Effectiveness								
Cumulative Probability of Success *		0%	10%	20%	30%	42%	50%	60%	80%
	0%	−$12,277,791	−$12,277,791	−$12,277,791	−$12,277,791	−$12,277,791	−$12,277,791	−$12,277,791	−$12,277,791
	10%	−$31,211,533	−$25,755,817	−$19,995,579	−$13,908,505	−$6,138,272	−$654,625	$6,567,374	$22,358,658
	13.7%	−$33,742,589	−$26,242,163	−$18,323,086	−$9,954,679	$727,706	$8,266,532	$18,195,214	$39,904,805
	20%	−$37,413,498	−$26,502,066	−$14,981,592	−$2,807,443	$12,733,023	$23,700,318	$38,144,315	$69,726,883
	30%	−$42,481,010	−$26,113,862	−$8,833,150	$9,428,073	$32,738,772	$49,189,714	$70,855,711	$118,229,562
	40%	−$46,973,332	−$25,150,468	−$2,109,519	$22,238,778	$53,319,710	$75,254,300	$104,142,295	$167,307,430
	50%	−$51,099,995	−$23,821,415	$4,979,771	$35,415,143	$74,266,308	$101,684,545	$137,794,539	$216,750,958
	60%	−$54,966,804	−$22,232,508	$12,328,915	$48,851,362	$95,472,759	$128,374,644	$171,706,637	$266,454,340
	70%	−$58,636,099	−$20,446,087	$19,875,573	$62,485,094	$116,876,724	$155,262,256	$205,816,248	$316,355,235
	80%	−$62,148,326	−$18,502,598	$27,579,300	$76,275,895	$138,437,758	$182,306,938	$240,082,929	$366,413,199
	90%	−$65,531,521	−$16,430,077	$35,412,058	$90,195,727	$160,127,824	$209,480,651	$274,478,640	$416,600,194
	100%	−$68,806,089	−$14,248,929	$43,353,443	$104,224,187	$181,926,516	$236,762,991	$308,982,979	$466,895,817

* Probabilities of success for the main developmental stages, which cumulate to the overall probability of success, were adjusted in the two-way sensitivity analysis. This resulted in rNPVs that are approximate to those in the base–case rNPV model. For example, for a 60% effectiveness at a 13.7% cumulative probability of success, the two-way analysis yields an rNPV of $18,195,214 compared to $18,325,117 in the base–case model.

## Data Availability

Data are contained within the article and Supplementary Materials.

## Supplementary Materials

The following supporting information can be downloaded at: https://www.mdpi.com/article/10.3390/jmahp13010001/s1.

## Glossary of Terms

**Arteriovenous Fistula:** An arteriovenous fistula is an arteriovenous vascular access created by surgically connecting a vein to an artery [9]. **Central Venous Catheter:** This is an indwelling catheter inserted into a central vein such as the subclavian vein to reach the inferior vena cava, superior vena cava or right atrium [60].

## List of Abbreviations

**AVG:** Arteriovenous graft; **COGS:** Cost of goods sold; **CEM:** Cost-effectiveness modeling; **CUA:** Cost-utility analysis; **CVC:** Central venous catheter; **DF-HTA:** Development-focused Health Technology Assessment; **ePTFE:** Expanded polytetrafluoroethylene; **ICER:** Institute for Clinical and Economic Review; **INMB:** Incremental net monetary benefit; **OCOC:** Opportunity Cost of Capital; **RCT:** Randomized controlled trial; **rNPV:** Risk-adjusted net present value; **SG&A:** Selling, general and administrative expenses; **WACC**: Weighted average cost of capital

## Appendix A

1. Additional details on the Decision Analytic Model

Deriving Utilities for the Cost–Utility Analysis

Wyld et al. [23] estimated the mean utility for hemodialysis as 0.69 (95% CI: 0.59–0.80). This was used as the “base” utility for the functional AVG state for both ePTFE and Smart AVGs. To estimate the utility of the CVC state, the study by Xue et al. [27] was referenced. In their study [27], resultant state utilities from published literature were used, and from this, the CVC state was assigned a utility that was 4.21% less than that of the AVG state. This percentage reduction was similarly applied to the base AVG utility of 0.69 to yield a utility value of 0.66 for the CVC state. The utility value for the failed AVG state was estimated by applying a disutility of 0.09, giving 0.60 (this value was an average of 0.08 and 0.1, disutility values for percutaneous and surgical interventions according to published literature [26,27]). A disutility of sepsis was applied to the AVG, failed AVG and CVC states. The monthly probability of sepsis was derived from the study by Locham et al. [25], which examined 870,571 patients initiating hemodialysis from 2006 to 2014 from the United States Renal Data System (USRDS). From this study, the incidence rate of sepsis for patients with AVG was 11.49 per 100 person-years, and that for those with CVC was 13.86 per 100 person-years. This was converted to a per person-year rate and used as the yearly probability of sepsis. The yearly probabilities were further converted to monthly probability using the formula: 1-(1-yearly probability)^1/12^. The monthly probability of sepsis for AVG was hence 0.0101, and that for CVC was 0.0124. The final disutility values were derived as a product of the monthly probability of sepsis for each health state, and the disutility of sepsis, which was 0.6 according to the literature [24,27]. This resulted in disutility values of 0.00606 and 0.00744 for the functional AVG and CVC states, respectively. The functional AVG state disutility was also applied to the failed AVG state. Eventually, the utility for each state was as follows: AVG state: 0.6839; Failed AVG state: 0.5939; and CVC state: 0.6526.

Transition probabilities (caveat)

It was assumed that the transition from a functional graft to death would remain the same for smart AVGs as for ePTFE, as it is most likely that any benefit the smart AV graft would offer in improving the quality and length of life would be through the prevention of graft failure.

Inflation: Costs were inflated to 2023 using Personal Health Care (PHC) expenditure indices [61] and Personal consumption expenditures (PCE) indices published by the US Bureau of Economic Analysis [62]. Although ICER recommends using annual indices for the calculation of inflation, the 2023 first quarter PCE index (in the absence of an annual 2023 PCE index at the time of conduct of the study) was used in inflating costs.

Model Validation (External validation)

As a form of model validation, the secondary patency (i.e., patency after graft placement until graft abandonment, transplant, or death, including intervening interventions [63,64]) of ePTFE graft in the model was compared to that from the meta-analysis by Halbert et al. [15]. From the CUA model, the secondary patency at 6 months, 12 months, and 24 months were 81.2%, 70.0%, and 52.1%, respectively, compared to those reported by Halbert et al. [15], which were 80% (95% CI: 75–84%), 70% (95% CI: 64–75%), and 54% (95% CI: 47–61%).

**Table A1:** An example of rNPV calculation for the smart AVG with a target annual COC/WACC * of 10.4% and an assumed smart AVG effectiveness level of 60%.

	Item	Estimate
Revenues	Unit Price	$22,455
	Cost of Goods Sold/device	$100
	SG&A Costs as a percentage of Sales Revenue	30%
	Total Forecast Unit Sales 2035–2044	130,368
	Net revenue if successful (net of COGS and SG&A)	$2,036,178,122
	Overall Probability of Success	13.7%
	Probability Success Weighted Net Revenue	$279,930,331
	Discount Factor for Future Sales	81.6%
	Discounted Total Revenue	$51,607,383
Cost of Development	Probability Weighted Development Costs	$48,768,344
	Discount Factor for Future Development Costs	31.4%
	Discounted Total Costs	$33,412,169
Risk-adjusted Net Present Value		$18,195,214

* COC: Cost of Capital WACC: Weighted average cost of capital.
