# Supplementary material for: Assessing the Value of Further Investment in R&D Using Mixed Methods: A Case Study of Biosensor-Integrated Arteriovenous Grafts
Source: J Mark Access Health Policy. 2025 Jan 15;13(1):1. doi: 10.3390/jmahp13010001 (PMC11755449; doi:10.3390/jmahp13010001)
Supplement: Supplementary file 1 [file jmahp-13-00001-s001.zip › jmahp-3345013-supplementary.pdf]

SUPPLEMENTARY FILES

1. Further details on the Decision Analytic model

Deriving Utilities for the Cost-Utility Analysis

Wyld et al.[1] estimated the mean utility for hemodialysis as 0.69 (95% CI: 0.59-0.80). This was used as the "base" utility for the functional AVG state for both ePTFE and Smart AVGs. To estimate the utility of the CVC state, the study by Xue et al.[2] was referenced. In their study,[2] resultant state utilities from published literature were used and from this, the CVC state was assigned a utility that was 4.21% less than that of the AVG state. This percentage reduction was similarly applied to the base AVG utility of 0.69 to yield a utility value of **0.66** for the CVC state. The utility value for the failed AVG state was estimated by applying a disutility of 0.09 giving 0.60 (this value was an average of 0.08 and 0.1, disutility values for percutaneous and surgical interventions according to published literature [2,3]). A disutility of sepsis was applied to the AVG, failed AVG and CVC states. The monthly probability of sepsis was derived from the study by Locham et al. [4] which examined 870,571 patients initiating hemodialysis from 2006 to 2014 in the United States Renal Data System (USRDS). From this study, the incidence rate of sepsis for patients with AVG was 11.49 per 100 person-years, and that for those with CVC was 13.86 per 100 person-years. This was converted to a per person-year rate and used as the yearly probability of sepsis. The yearly probabilities were further converted to monthly probability using the formula: $1-(1\text{-yearly probability})^{1/12}$. The monthly probability of sepsis for AVG was hence 0.0101 and that for CVC was 0.0124. The final disutility values were derived as a product of the monthly probability of sepsis for each health state and the disutility of sepsis which is 0.6 according to the literature.[2,5] This resulted in disutility values of 0.00606 and 0.00744 for the functional AVG and CVC states respectively. The functional AVG state disutility was also applied to the failed AVG state.

Eventually, the utility for each state was as follows: AVG state**: 0.6839;** Failed AVG state: **0.5939**; and CVC state: **0.6526**.

Transition probabilities (caveat)

It was assumed that the transition from a functional graft to death would remain the same for smart AVGs as for ePTFE as it is most likely that any benefit the smart AV graft would offer in improving the quality and length of life would be through the prevention of graft failure.

Inflation: Costs were inflated from 2015 (reference year for costs by Al-Balas et al.[6]) to 2023 using Personal Health Care (PHC) expenditure indices [7] and Personal consumption expenditures (PCE) indices published by the US Bureau of Economic Analysis.[8] Although ICER recommends using annual indices for the calculation of inflation, the 2023 first quarter PCE index (in the absence of an annual 2023 PCE index at the completion of the study) was used in inflating costs from Al-Balas et al.[6] .

Model Validation (External validation)

As a form of model validation, the secondary patency, (i.e. patency after graft placement until graft abandonment, transplant or death including intervening interventions [9,10]) of ePTFE graft in the model was compared to that from the meta-analysis by Halbert *et al.*[11] From the model, the secondary patency at 6 months, 12 months and 24 months were 81.2%, 70.0%, and 52.1% respectively, compared to those reported by Halbert *et al.*[11] which were 80% (95% CI: 75% - 84%), 70% (95% CI: 64% - 75%), and 54% (95% CI: 47%-61%).

2. Details on rNPV analysis

Estimating AV graft Market size for the rNPV analysis

Market size for AV grafts was estimated using data from the US Renal Data System (USRDS) annual reports.[12] The mean incidence of hemodialysis cases per year from 2000-2019 was calculated to be 343.7 cases/million persons in the US population. From the reports, at the initiation of hemodialysis, most patients have a central venous catheter but transition to an AVG or AVF by 18 months. Recent reports show that after 18 months, about 16.9% (16.8% for those starting in 2017 and 17% for 2018) of patients initiating hemodialysis eventually end up with an AVG. Using the average incidence of hemodialysis and US population projections by the OECD,[13] hemodialysis cases for 2035-2045 were estimated. Of these estimates, 16.9% represented the AVG market size for each respective year. The market penetration was assumed to be 15% for the first year of sales and assumed to increase by 15 percentage points each subsequent year till 2039, after which the market penetration was assumed to stay at 75% till 2044.

SUPPLEMENTARY TABLES

Supplementary Table A. Probabilities of success in base-case and two-way sensitivity analysis

**Base-case analysis**			**Two-way sensitivity analysis**		
Stage of development or approval	***Probability of stage success***	***Cumulative probability of success (at end of the stage)***	***Stage***	***Probability of stage success***	***Cumulative probability of success***
Concept Development	77.8%	77.8%	Concept Development	78.03%	78.0%
Clinical Unit Development	77.8%	60.5%	Clinical Unit Development	78.03%	60.9%

IDE Application	77.8%	47.0%	IDE Application	78.03%	47.5%
Clinical Safety Study	48.0%	22.6%	Clinical Safety Study 1	78.03%	37.1%
Pivotal Clinical Trial	75.7%	17.1%	Clinical Safety Study 2	78.03%	28.9%
PMA Approval Process	80.5%	13.7%	Clinical Safety Study 3	78.03%	22.6%
			Pivotal Clinical Trial	78.03%	17.6%
			PMA Approval Process	78.03%	13.7%

Supplementary Table B. Parameters for the CUA

	PARAMETER	VALUE	DATA SOURCE(S)	COMMENTS
**Costs (2023 $)**	AVG (ePTFE placement)	$4,641.19	Al-Balas *et al*.[6]	Reimburseme nt prices. Includes professional and facility fees.
	Intervention/Revision after non-maturation of graft	$4,593.26		
	CVC placement	$896.97		
	Monthly cost for failed AVG state (monthly cost of interventions for failed AVG)	$647.62	Al-Balas *et al*.[6]	Source provides median annual vascular access related cost for AVG for 2015. Inflation applied and yearly cost

				converted to monthly cost.
	Monthly cost for CVC state	$2,730.18	Al-Balas *et al*.[6]	Median annual vascular access-related cost (Percutaneous and Surgical procedures, and hospitalisation for bacteraemia, 2015)
**UTILITIES**	Functioning AVG (both ePTFE and Smart Graft)	0.6839	Wyld *et al*.[1], Scheetz *et al*.,[5] Locham *et al*.,[4]	-Mean Utility estimate from Wyld et al. (0.6900)[3] -A disutility of 0.0061 applied for sepsis [4,5]
	Failed AVG (both ePTFE and Smart Graft)	0.5939	Brothers *et al*.,[3] Scheetz *et al*.,[5]	-Utility of functioning AVG (0.6839)

			Locham *et al.*[4]	-Disutility of 0.09 applied for percutaneous and surgical interventions[6]
	CVC	0.6526	Xue *et al.*[2], Brother *et al.*[3], Scheetz *et al.*[5]	-4.21% of functioning AVG utility deducted as disutility for having CVC (0.6600)[7]<br>-A disutility of 0.0074 applied for sepsis [4,5]
**TRANSITION PROBABILITIES**	Death after procedure	0.022	Leermakers *et al.*[14]	**For the Decision tree, probabilities, costs, and outcomes were assumed to be the same for both ePTFE*
	Death during maturation	0.054		
	Mature/functional (after maturation)	0.772		
	Death after non-maturation	0.011		
	New Access/CVC after non-maturation	0.065		

	Functionality after intervention (post-non-maturation)	0.076		*and Smart AV graft arm.*
	Functioning graft state to Continued function**	0.932 (for ePTFE grafts)	Leermakers *et al.*[14]	***For the Markov model, besides the transition from "function to failure" and "continued function" all other transitions and corresponding probabilities and costs were assumed to be the same for ePTFE and Smart AV graft.*
	Function to failure**	0.057 (for ePTFE grafts)		
	Function to death	0.011		
	Failed graft state to Continued failure	0.280		
	Failure to functioning	0.533		
	Failure to CVC	0.120		
	Failure to death	0.067		
	CVC State to Continued CVC	0.974	Xue *et al.*[2]	
	CVC to death	0.026		

Supplementary Table C. Market size estimation

Year	***Market size estimate***	***Estimated Market Penetration***	***Estimated Number of Sales***	***Sources***
2035	21,195	15%	*3179*	Current market size estimates are based on annual reports from the US Renal Data System.[12] OECD population projections were used in addition to current and past rates of AVG use from USRDS to estimate future market size.[13] (Details in *Supplementary material*)<br><br>Source for market penetration estimates: Based on assumptions
2036	21,300	30%	*6390*	
2037	21,403	45%	*9631*	
2038	21,504	60%	*12902*	
2039	21,602	75%	*16201*	
2040	21,698	75%	*16274*	
2041	21,793	75%	*16344*	
2042	21,885	75%	*16414*	
2043	21,976	75%	*16482*	
2044	22,066	75%	*16550*	
Total			*130,368*	

REFERENCES

1. Wyld M, Morton RL, Hayen A, Howard K, Webster AC. A Systematic Review and Meta-Analysis of Utility-Based Quality of Life in Chronic Kidney Disease Treatments. *PLoS Med*. 2012;9(9):e1001307. doi:10.1371/JOURNAL.PMED.1001307

2. Xue H, Lacson E, Wang W, Curhan GC, Brunelli SM. Choice of Vascular Access among Incident Hemodialysis Patients: A Decision and Cost-Utility Analysis. *Clinical Journal of the American Society of Nephrology*. 2010;5(12):2289-2296. doi:10.2215/CJN.03210410

3. Brothers TE, Cox MH, Robison JG, Elliott BM, Nietert P. Prospective decision analysis modeling indicates that clinical decisions in vascular surgery often fail to maximize patient expected utility. *Journal of Surgical Research*. 2004;120(2):278-287. doi:10.1016/J.JSS.2004.01.004

4. Locham S, Naazie I, Canner J, Siracuse J, Al-Nouri O, Malas M. Incidence and risk factors of sepsis in hemodialysis patients in the United States. *J Vasc Surg*. 2021;73(3):1016-1021.e3. doi:https://doi.org/10.1016/j.jvs.2020.06.126

5. Scheetz MH, Bolon MK, Postelnick M, Noskin GA, Lee TA. Cost-effectiveness analysis of an antimicrobial stewardship team on bloodstream infections: a probabilistic analysis. *Journal of antimicrobial chemotherapy*. 2009;63(4):816-825.

6. Al-Balas A, Lee T, Young CJ, Kepes JA, Barker-Finkel J, Allon M. The clinical and economic effect of vascular access selection in patients initiating hemodialysis with a catheter. *Journal of the American Society of Nephrology*. 2017;28(12):3679-3687. doi:10.1681/ASN.2016060707/-/DCSUPPLEMENTAL

7. AHRQ. 1.1 Using Appropriate Price Indices for Expenditure Comparisons. Accessed May 20, 2023. https://meps.ahrq.gov/about_meps/Price_Index.shtml

8. BEA Interactive Data Application. Accessed May 20, 2023. https://apps.bea.gov/iTable/?reqid=19&step=2%23reqid%3D19&step=2&isuri=1&1921=survey#eyJhcHBpZCI6MTksInN0ZXBzIjpbMSwyLDMsM10sImRhdGEiOltbIkNhdGVnb3JpZXMiLCJTdXJ2ZXkiXSxbIk5JUEFfVGFibGVfTGlzdCIsIjY0Il0sWyJGaXJzdF9ZZWFyIiwiMjAyMSJdLFsiTGFzdF9ZZWFyIiwiMjAyMyJdLFsiU2NhbGUiLCIwIl0sWyJTZXJpZXMiLCJRIl1dfQ==

9. Allemang MT, Schmotzer B, Wong VL, et al. Arteriovenous grafts have higher secondary patency in the short term compared with autologous fistulae. *The American Journal of Surgery*. 2014;208(5):800-805. doi:10.1016/J.AMJSURG.2014.01.010

10. Sidawy AN, Gray R, Besarab A, et al. Recommended standards for reports dealing with arteriovenous hemodialysis accesses. *J Vasc Surg*. 2002;35(3):603-610. doi:10.1067/mva.2002.122025

11. Halbert RJ, Nicholson G, Nordyke RJ, Pilgrim A, Niklason L. Patency of ePTFE Arteriovenous Graft Placements in Hemodialysis Patients: Systematic Literature Review and Meta-Analysis. *Kidney360*. 2020;1(12):1437. doi:10.34067/KID.0003502020

12. United States Renal Data System U. Annual Data Report | USRDS. *USRDS*. Published online 2021. Accessed March 22, 2022. https://adr.usrds.org/2021/end-stage-renal-disease/4-vascular-access

13. Organisation for Economic Co-operation and Development O. Population projections. Accessed November 11, 2022. https://stats.oecd.org/Index.aspx?DataSetCode=POPPROJ#

14. Leermakers J, Bode AS, Vaidya A, van der Sande FM, Evers S, Tordoir JHM. Cost-effectiveness of vascular access for haemodialysis: arteriovenous fistulas versus arteriovenous grafts. *European journal of vascular and endovascular surgery*. 2013;45(1):84-92.
